# A practical guide to the implementation of AI in orthopaedic research‐Part 4: Prerequisites for a successful orthopedics AI‐driven project in terms of interdisciplinary collaboration, data management, ethical approval and technology

**DOI:** 10.1002/jeo2.70863

**Published:** 2026-07-28

**Authors:** Umile Giuseppe Longo, Mario Merone, Emiliano Schena, Benedetta Bandini, Guido Nicodemi, Bálint Zsidai, Ann‐Sophie Hilkert, Eric Hamrin Senorski, Alberto Grassi, Christophe Ley, Elmar Herbst, Michael T. Hirschmann, Sebastian Kopf, Romain Seil, Thomas Tischer, Robert Feldt, Kristian Samuelsson, Felix C. Oettl

**Affiliations:** ^1^ Fondazione Policlinico Universitario Campus Bio‐Medico Roma Italy; ^2^ Research Unit of Orthopaedic and Trauma Surgery, Department of Medicine and Surgery Università Campus Bio‐Medico di Roma Roma Italy; ^3^ Department of Engineering University Campus Bio‐Medico di Roma Rome Italy; ^4^ Sahlgrenska Sports Medicine Center Gothenburg Sweden; ^5^ Department of Orthopaedics, Institute of Clinical Sciences, Sahlgrenska Academy University of Gothenburg Gothenburg Sweden; ^6^ Department of Computer Science and Engineering Chalmers University of Technology Gothenburg Sweden; ^7^ Department of Health and Rehabilitation, Institute of Neuroscience and Physiology, Sahlgrenska Academy University of Gothenburg Gothenburg Sweden; ^8^ Sportrehab Sports Medicine Clinic Gothenburg Sweden; ^9^ IIa Clinica Ortopedica e Traumatologica, IRCCS Istituto Ortopedico Rizzoli Bologna Italy; ^10^ Department of Mathematics University of Luxembourg Esch‐sur‐Alzette Luxembourg; ^11^ Department of Trauma, Hand and Reconstructive Surgery University Hospital Münster Münster Germany; ^12^ Department of Orthopedic Surgery and Traumatology, Head Knee Surgery and DKF Head of Research Kantonsspital Baselland Bottmingen, Bruderholz Switzerland; ^13^ Center of Orthopaedics and Traumatology University Hospital Brandenburg a.d.H., Brandenburg Medical School Theodor Fontane Brandenburg a.d.H. Germany; ^14^ Faculty of Health Sciences Brandenburg Brandenburg Medical School Theodor Fontane Brandenburg a.d.H. Germany; ^15^ Department of Orthopaedic Surgery Centre Hospitalier Luxembourg and Luxembourg Institute of Health Luxembourg Luxembourg; ^16^ Department of Orthopaedic Surgery University Medicine Rostock Rostock Germany; ^17^ Department of Orthopaedics Sahlgrenska University Hospital Mölndal Sweden; ^18^ Department of Orthopedic Surgery University Hospital Balgrist Zurich Switzerland

**Keywords:** artificial intelligence, collaboration, management, structure

## Abstract

Translating artificial intelligence (AI) research in orthopedics from proof‐of‐concept studies into production‐grade clinical systems requires the systematic satisfaction of four prerequisite domains: interdisciplinary team architecture, technical data management, ethical and regulatory governance and production‐grade technology and deployment infrastructure. Despite a tenfold increase in orthopedic AI publications, fewer than 6% of studies reach routine clinical deployment, reflecting persistent gaps in each of these domains. This article provides a technically rigorous, evidence‐based framework organized around these four pillars. The interdisciplinary team may be structured using a product‐centric topology that decouples stream‐aligned clinical teams from platform infrastructure teams, following Huffman et al.'s six‐step AI project lifecycle: obtain/curate/label data; establish a reference standard; develop the model; evaluate performance; externally validate and iteratively reinforce until clinical implementation is viable. Data management requires data extraction protocols, integration for bulk exports and a multi‐component de‐identification pipeline. A multi‐stage Institutional Review Board framework governs ethical oversight, scaling from Exempt review for retrospective de‐identified studies to Full Board Review with prospective validation and mandatory human‐override mechanisms for interventional deployment. Responsible clinical deployment requires a multi‐layer Clinical Machine Learning Operations framework, implementing privacy‐preserving deployment, clinical observability, compliance audit trails and human‐in‐the‐loop governance. Model drift has to be monitored with a degradation threshold triggering mandatory human review.

**Level of Evidence:**

Level V.

**Abbreviations**
AUC: area under the curve
BioBERT‐CRF: Bidirectional Encoder Representations from Transformers for Biomedical Text, with Conditional Random Field
CNN: convolutional neural network
DICOM: Digital Imaging and Communications in Medicine
EHR: electronic health record
ETL: extract‐transform‐load
FHIR: Fast Healthcare Interoperability Resources
FL: federated learning
FPR: false positive rate
GAN: Generative Adversarial Network
GDPR: General Data Protection Regulation
Grad‐CAM: Gradient‐weighted Class Activation Mapping
HIPAA: Health Insurance Portability and Accountability Act
HITL: human‐in‐the‐loop
IRB: Institutional Review Board
LIME: Local Interpretable Model‐agnostic Explanations
LLM: large language model
LoRA: Low‐Rank Adaptation
LUT: look‐up table
MLOps: Machine Learning Operations
PACS: Picture Archiving and Communication System
PCCP: Predetermined Change Control Plan
PHI: Protected Health Information
RAG: Retrieval‐Augmented Generation
RIS: Radiology Information System
ROC: receiver operating characteristic
SaMD: Software as a Medical Device
SHAP: SHapley Additive exPlanations
SMART: Substitutable Medical ApplicAtions, Reusable Technologies
SR: Structured Report (DICOM)
ViT: vision transformer
XAI: explainable artificial intelligence
XGBoost: extreme gradient boosting
XNAT: eXtensible Neuroimaging Archive Toolkit

## INTRODUCTION

### The scale of the problem

Orthopedic surgery generates substantial volumes of multimodal data: plain radiographs, cross‐sectional computed tomography (CT) and magnetic resonance imaging (MRI) studies, operative notes, implant registry records, outcome questionnaires and postoperative wearable sensor streams. The increasing density and heterogeneity of these data have rendered manual analysis insufficient for detecting population‐level patterns relevant to diagnosis, prognosis, surgical planning and treatment optimization [16, 17, 18, 20, 21, 26].

Yet the transition from isolated research studies with high in‐sample accuracy to robust, governable clinical systems remains rare. A tenfold increase in orthopedic artificial intelligence (AI) publications over the past decade has not produced a proportional increase in clinical deployment: fewer than 6% of AI studies in the United Kingdom's National Health Service (NHS) reach routine deployment [27]. The barriers are not primarily algorithmic. They are engineering, organizational, regulatory and cultural: the absence of interoperable data pipelines; inadequate de‐identification infrastructure; unscaled ethical frameworks; deployment architectures that do not satisfy regulatory standards and the absence of post‐deployment monitoring capable of detecting model drift before it causes patient harm.

### The four‐phase AI project lifecycle

A practical AI‐driven orthopedic research project advances through four sequential phases:

1. Conceptualization: Definition of the clinical problem and measurable target variable; assessment of data availability; identification of the optimal model family; formation of the interdisciplinary team; and initial ethical and regulatory scoping.
2. Data acquisition and preparation: Extraction of imaging and clinical data from institutional systems (Picture Archiving and Communication System [PACS], electronic health record [EHR], registries); de‐identification to applicable standards; curation, quality control and annotation to generate ground‐truth labels; and assembly of stratified training, validation and test datasets.
3. AI application: Selection and training of an architecture matched to the data modality and task; management of class imbalance; multi‐metric performance evaluation; external validation on independent datasets and reporting according to standards.
4. Translation to clinical practice: Containerized deployment within hospital infrastructure; integration with PACS and EHR workflows via Digital Imaging and Communications in Medicine (DICOM) Structured Report (SR) and Health Level Seven (HL7) interfaces; regulatory clearance as Software as a Medical Device (SaMD); implementation of Clinical Machine Learning Operations (MLOps) governance and ongoing performance monitoring and model drift management.

**Figure 1:**
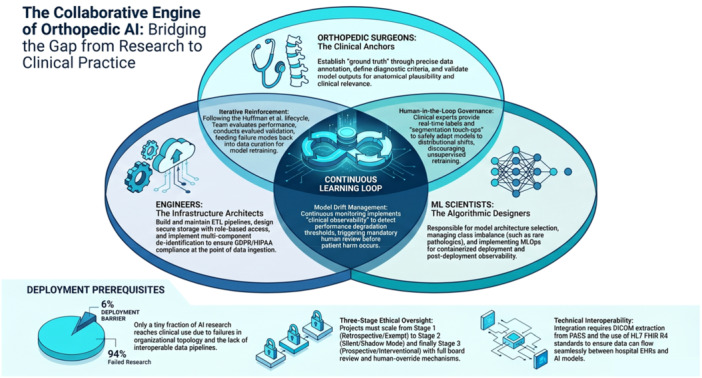
Schematic overview of the phases of an AI‐driven orthopedic research project that must be satisfied. AI, artificial intelligence; DICOM, Digital Imaging and Communications in Medicine; EHR, electronic health record; ETL, extract‐transform‐load; GDPR, General Data Protection Regulation; HIPAA, Health Insurance Portability and Accountability Act; HL7 FHIR R4, Health Level Seven Fast Healthcare Interoperability Resources R4; ML, machine learning; MLOps, Machine Learning Operations; PACS, Picture Archiving and Communication System.

Each phase imposes technical and organizational requirements that, when neglected, produce systems that are either methodologically invalid or operationally non‐deployable. The capital costs of real‐time AI processing infrastructure in hospital environments are massive, underscoring the need for rigorous prerequisite planning before committing institutional resources (Figure 1).

## PILLAR A: INTERDISCIPLINARY COLLABORATION AND TEAM TOPOLOGIES

### The six‐step project lifecycle

Successful orthopedic AI projects require institutional commitment and a multidisciplinary team in which clinical and technical expertise are functionally integrated—not merely co‐listed on a grant application [7]. Huffman et al. define a six‐step operational lifecycle that governs team activity across all four phases of the project:

1. Obtain, curate and label data.
2. Establish a reference standard (ground truth).
3. Develop the AI model.
4. Evaluate model performance with validated metrics.
5. Conduct external validation on an independent dataset.
6. Iteratively reinforce and evaluate the model until clinical implementation is feasible.

This lifecycle is not sequential in the project‐management sense; Steps 4–6 form a feedback loop that may restart at Step 1 when external validation reveals systematic failure modes. Planning for iteration is therefore a prerequisite, not an afterthought.

### Team topology: Product‐centric versus project‐centric

A critical but underappreciated organizational decision is the choice between project‐centric and product‐centric team topologies. Project‐centric teams dissolve upon study completion, producing orphaned models with no operational owner. Product‐centric topologies treat the clinical AI system as a living product requiring ongoing maintenance and are strongly favoured for any deployment‐targeted project.

### Role definitions and task responsibilities

#### Orthopedic surgeons and radiologists (clinical domain experts)

Clinical domain experts bear responsibility for tasks that, if delegated to non‐clinical personnel, introduce systematic labelling errors or clinically invalid model objectives. Their primary functions include:

##### Ground truth annotation

Generating reference‐standard segmentation masks, bounding box labels, fracture classifications and diagnostic ground truth across the dataset.

##### Reference standard establishment

Defining the diagnostic criteria against which all model outputs are evaluated. Inter‐annotator agreement (Cohen's *κ*) must be computed and reported; a threshold may be set before model training proceeds, as below the threshold, label noise introduces a performance ceiling that cannot be overcome by architectural improvements.

##### Clinical validation

Evaluating model outputs for anatomical plausibility, classification consistency with established taxonomies (e.g., garden classification for femoral neck fractures; AO classification for long bone fractures) and clinical relevance. This is distinct from statistical performance evaluation and requires domain expertise that is not automatable. Structured review sessions with machine learning (ML) scientists—using explainability tools to inspect model attention on confusing cases—are an operationally validated approach to iterative model refinement [14].

##### Informed consent and patient‐facing communication

Designing the patient transparency statement for deployment, explaining the role of AI in the clinical pathway and the specific risks unique to the technology. This requires clinical understanding of what the model does and does not do.

##### Workflow integration assessment

Evaluating the operational fit of AI outputs within existing clinical workflows prior to deployment, including assessment of alert fatigue risk from false‐positive rates.

#### Engineers

Engineers construct and maintain the technical infrastructure that enables data flow from source clinical systems to AI training and inference environments. Their responsibilities span the full data lifecycle:

##### Extract‐transform‐load (ETL) pipeline design and operation

Building ETL pipelines that ingest files, apply de‐identification, validate data quality and deliver structured datasets to training environments.

##### De‐identification and compliance infrastructure

Operating de‐identification pipelines that satisfy General Data Protection Regulation (GDPR) and/or Health Insurance Portability and Accountability Act (HIPAA) requirements at the point of data ingestion, prior to any model training or analysis.

##### Secure storage architecture

Configuring on‐premise or hybrid cloud storage with encryption in transit and at rest, role‐based access controls and tamper‐evident audit logging for all data access events.

##### Data quality governance

Enforcing standardized quality checks: modality code mismatch tolerance to prevent corrupted labels; DICOM header metadata validation and voxel spacing normalization across multi‐vendor datasets.

##### DICOM preprocessing automation

Implementing preprocessing pipelines that run automatically on all new data ingested into the training environment.

#### ML scientists

ML scientists are responsible for the algorithmic components, experiment infrastructure and deployment engineering:

##### Model architecture selection

Matching the appropriate architectural family (convolutional neural network [CNN], vision transformer (ViT), large language model [LLM], segmentation network) to the data modality, dataset scale, inference speed requirements and interpretability constraints.

##### Class imbalance management

Implementing augmentation strategies, cost‐sensitive learning and synthetic data generation to address systematic underrepresentation of positive cases in orthopedic datasets.

##### Experiment tracking and reproducibility

Logging hyperparameters, dataset hashes, model artefacts and metric trajectories. Dataset versions may be identified to ensure that training provenance is reconstructable from artefact metadata alone.

##### Evaluation metric design

Selecting metrics appropriate to the clinical task and reporting them with 95% confidence intervals from bootstrapped test sets.

##### MLOps deployment

Packaging trained models in containers, integrating them with hospital middleware via REST application programming interface (APIs), configuring DICOM SR output for PACS return paths and establishing Clinical MLOps monitoring pipelines for post‐deployment observability.

##### LLM adaptation strategy

For natural language processing (NLP) components, selecting between full fine‐tuning (maximum expressivity but high risk of catastrophic forgetting on small orthopedic datasets), Low‐Rank Adaptation (LoRA) or Retrieval‐Augmented Generation (RAG) (near‐zero hallucination rate but higher computational latency—unsuitable for real‐time intraoperative contexts).

### Iterative refinement and the continuous learning loop

The NeoMedSys platform case study (Liu et al., VIOLA‐AI) demonstrates the importance of building a continuous learning loop into the deployment architecture from the outset [10]. In this platform, clinical radiologists push newly ingested imaging studies to an annotation interface (NeoSeg), provide real‐time labels and segmentation touch‐ups, and these curated cases are used for near‐real‐time model retraining. Critically, this improvement required disciplined human oversight: Unsupervised retraining is explicitly discouraged in healthcare contexts because automated adaptation to distributional shift may introduce unvalidated behavioural changes with patient‐safety consequences.

## PILLAR B: DATA MANAGEMENT AND PREPROCESSING

### DICOM extraction from PACS systems

Clinical imaging data in orthopedics is stored in DICOM format within hospital PACS infrastructure. DICOM extraction is the first technical bottleneck in the data pipeline; traditional DICOM data management systems are inadequate for the scale and structural complexity required by ML algorithms, necessitating purpose‐built platforms incorporating search, automated annotation and efficient tagging functionalities [1].

Technical challenges in DICOM extraction present a significant and underestimated obstacle. Different scanner vendors (GE, Siemens, Philips) implement optional DICOM tags inconsistently, and hospitals use variable conventions for body part, laterality and acquisition parameters [2]. Cross‐vendor imaging variability causes domain shift in trained models: non‐uniform slice thickness and field‐of‐view distort CNN feature extraction layers, producing decreased classification precision and elevated false‐negative rates in under‐standardized imaging environments.

### HL7 FHIR R4 and clinical data interoperability

For structured clinical data—operative notes, patient demographics, comorbidities, outcome scores, implant records—HL7 FHIR (Fast Healthcare Interoperability Resources) R4 has become the dominant interoperability standard, utilizing JSON, XML and REST APIs to achieve semantically interoperable exchange [5]. Combined, these create a machine‐readable semantic layer that is essential for constructing AI‐ready datasets from heterogeneous EHR systems.

SMART on FHIR (Substitutable Medical Applications, Reusable Technologies) creates open standards and APIs that allow applications to be ‘plugged in’ to different EHR systems without custom development for each vendor—the clinical AI equivalent of a universal adapter [12]. Interoperability mandates such as the U.S. 21st Century Cures Act further enforce adoption of FHIR‐based patient data access.

### De‐identification pipeline architecture

De‐identification must be achieved at the point of data ingestion, before any analytical use. Retrospective access to de‐identified secondary data is the most common ethical pathway for Stage 1 AI studies, and the technical quality of de‐identification determines whether the resulting dataset is permissible for institutional use and appropriate for training a model without introducing re‐identification risk.

#### DICOM metadata de‐identification

DICOM headers contain Protected Health Information (PHI) distributed across hundreds of potentially populated tags: patient name, date of birth, accession number, institution name, referring physician, device serial number, acquisition date and many others. The DICOM Basic Attribute Confidentiality Profile (PS 3.15) defines a standardized attribute‐level approach for PHI removal while preserving the technically essential acquisition parameters that AI models require. Python‐GDCM implements this profile programmatically, stripping identifying tags while preserving critical technical acquisition parameters.

Preserving these parameters is essential because systematic differences in acquisition technique across scanners introduce quantifiable confounds. A model trained on images from a single scanner configuration may fail to generalize to studies acquired at different kVp settings if this variation is not present in the training set.

#### Free‐text clinical note de‐identification

Unstructured clinical text—operative reports, radiology reads, discharge summaries, clinic notes—contains PHI embedded within variable‐length prose that regular expression matching alone cannot reliably identify. A two‐component hybrid pipeline is required:

1. Regular expression pattern matching: Removes structured PHI patterns that follow consistent formats: date strings, medical record numbers, accession numbers, telephone numbers and email addresses.
2. BioBERT‐CRF (Bidirectional Encoder Representations from Transformers for Biomedical text, with Conditional Random Field output layer): Identifies and redacts person names, institutional references and geographic identifiers in clinical prose with context awareness—distinguishing, for example, a physician name from an anatomical structure name that happens to match a surname.

### Imaging preprocessing standards

Standardized preprocessing is a prerequisite for model generalizability across imaging equipment from different vendors and acquisition protocols. The absence of a preprocessing standard is a barrier to AI generalizability, as publicly shared datasets have used inconsistent techniques.

DICOM LUT application: The mandatory first preprocessing step is applying the DICOM look‐up table (LUT) to transform raw stored pixel values to clinical standard display pixel values without information loss. Most radiology images are presented to radiologists through this transformation; AI models trained without LUT application will learn from non‐clinical pixel distributions and fail to generalize to real‐world workflows [3].

Voxel resampling: For CT and MRI datasets, voxel spacing is resampled to isotropic 1 × 1 × 1 mm resolution using interpolation, eliminating slice‐thickness variability across acquisition protocols as a source of domain shift.

### Annotation platform selection and comparison

The annotation platform is the operational centre of the data preparation phase. Platform selection has direct consequences for annotation throughput, inter‐rater reliability, dataset scalability and collaborative workflow management (Table 1). The following platforms are in active use in orthopedic AI research:

**Table 1:** Comparison of annotation platforms used in orthopedic AI research.

Platform	Modality	Scale	Collaboration	PACS integration	Best use case
XNAT	Multi	Large	Yes	DICOMweb	Multicenter imaging repositories
ITK‐SNAP	CT/MRI	Medium	Minimal	No	Single‐site segmentation
3D Slicer	Multi	Small‐Med	Limited	Plugin	Complex 3D annotation
Labellerr	2D XR	Large	Yes	Via screenshot	High‐throughput detection labelling
MD.ai	MultieXtensible Neuroimaging Archive Toolkit	Large	Yes	Limited	Structured multi‐reader annotation
MedCAT	Text	Any	No	N/A	Clinical NLP concept extraction

Abbreviations: 2D, two‐dimensional; 3D, three‐dimensional; AI, artificial intelligence; CT, computed tomography; DICOM, Digital Imaging and Communications in Medicine; MRI, magnetic resonance imaging; N/A, not applicable; NLP, natural language processing; PACS, Picture Archiving and Communication System; XNAT, eXtensible Neuroimaging Archive Toolkit; XR, X‐ray.

XNAT (eXtensible Neuroimaging Archive Toolkit): An open‐source web‐based platform for archiving, managing and sharing medical images and associated study data, designed specifically for large multicenter research repositories. XNAT includes the OHIF Viewer with DICOMweb integration and supports contour‐based and mask‐based ROI creation, a ‘smart CT’ paintbrush tool, and integration of NVIDIA AIAA for AI‐assisted annotation that reduces manual segmentation burden. XNAT is the appropriate choice for projects involving multiple institutions, large datasets requiring centralized quality control or studies requiring integration of imaging data with non‐imaging clinical metadata.

ITK‐SNAP: A widely used standalone application emphasizing semi‐automated segmentation using active contour algorithms. ITK‐SNAP reduces manual annotation workload compared to fully manual approaches. Limitations include minimal collaborative features and no built‐in workflow management, making it suboptimal for multi‐annotator projects requiring systematic case assignment and progress tracking.

Three‐dimensional (3D) Slicer: A comprehensive open‐source platform supporting multimodal imaging (CT, MRI, positron emission tomography) and 3D volumetric segmentation. 3D Slicer is appropriate for complex annotation tasks—where multi‐planar and volumetric context are essential. Its extensibility through a plugin architecture supports specialized orthopedic tasks. The steep learning curve and slower throughput make it less suitable for high‐volume two‐dimensional (2D) radiograph annotation workflows.

Labellerr: A HIPAA‐compliant cloud‐based object detection tool that operates on de‐identified radiographs captured as screenshots from PACS, with all patient identifiers excluded before export. It supports bounding box, polygon and keypoint annotation and collaborative multi‐annotator workflows with inter‐annotator agreement reporting. It is well‐suited to high‐throughput radiograph‐level labelling for detection and classification tasks.

MD.ai: A web‐based clinical annotation platform with a radiology‐native interface supporting multi‐reader structured annotation. MD.ai's collaborative architecture enables assignment of annotation tasks to specific team members, tracking of completion status and automated inter‐annotator agreement computation—features that are essential for maintaining annotation quality at scale.

MedCAT (Medical Concept Annotation Tool): Used for semi‐automated extraction of structured medical concepts from free‐text clinical notes, generating annotated training data for NLP models. MedCAT maps clinical text mentions to SNOMED CT, UMLS and custom ontology concepts with minimal supervision.

### Handling class imbalance

Class imbalance is a fundamental challenge in orthopedic AI model development. Common pathologies may be over‐represented relative to rare lesions or uncommon implant failure modes; within segmentation tasks, foreground pixels representing pathological structures are vastly outnumbered by background pixels. Failure to address imbalance leads to models biased toward the majority class, producing systematically high false‐negative rates for the clinically critical minority class [19, 28].

### Strategies by category

#### Data‐level interventions

Traditional augmentation: Geometric transformations (rotation ±15°, scaling, horizontal/vertical flipping), intensity augmentation (brightness α/β factors, Gaussian noise), elastic deformations and cutout regularization. These are implemented during training‐time data loading to prevent overfitting to augmentation artefacts.

SMOTified‐GANs: Hybrid approach combining Synthetic Minority Oversampling Technique (SMOTE) oversampling with Generative Adversarial Network (GAN)‐generated synthetic minority samples [23].

#### Algorithm‐level interventions

Focal loss (*γ* = 2): Down‐weights the contribution of easy, correctly classified majority‐class examples to the loss gradient, focusing training signal on difficult minority‐class cases.

Cost‐sensitive learning: Assigns class weights inversely proportional to class frequency in the cross‐entropy loss, penalizing misclassification of minority‐class examples more heavily.

Ensemble methods (stacking, soft voting): Combine predictions from multiple models trained on differently balanced bootstrap samples, reducing variance attributable to imbalance‐induced instability.

#### Dataset‐level interventions

Multicenter and demographically diverse dataset assembly: Incorporating patient populations spanning different races, sexes and age groups is essential for both algorithmic fairness and generalizability to real‐world clinical populations.

Standardized quality checks: Maintaining a low tolerance threshold for modality code mismatches prevents corrupted labels that are disproportionately likely to occur in minority‐class cases, compounding imbalance effects.

## RADIOMICS VERSUS END‐TO‐END DEEP LEARNING

A methodological choice that arises early in project design is whether to use handcrafted radiomic features or end‐to‐end deep learning for imaging analysis:

Radiomics extracts hundreds of mathematical features from manually or semi‐automatically segmented regions of interest (ROIs) (e.g., a vertebral body, a femoral head): first‐order intensity statistics (mean, entropy, kurtosis), second‐order texture matrices (gray‐level co‐occurrence matrix, gray level run length matrix) and higher‐order wavelet‐ and Laplacian of Gaussian‐filtered features. These numeric biomarkers are then supplied to classical ML classifiers (Extreme Gradient Boosting [XGBoost], Random Forest). Radiomics captures patterns not visible to the human eye—subtle textural changes in trabecular architecture predictive of impending fracture or malignancy—and produces interpretable, auditable feature sets.

End‐to‐end deep learning automatically extracts high‐dimensional hierarchical features in an end‐to‐end manner during training, mitigating the human bias introduced by manual ROI segmentation and feature selection.

The radiomics approach retains clinical value in small‐dataset contexts where end‐to‐end training is underpowered, or when interpretability of individual feature contributions is a regulatory or clinical requirement. Deep learning is preferable when datasets are sufficiently large and when predictive performance is the primary objective.

## PILLAR C: ETHICAL APPROVAL, GOVERNANCE AND REGULATORY COMPLIANCE

### The three‐stage Institutional Review Board (IRB) framework for AI research

The level of ethical oversight required for an orthopedic AI project scales directly with the degree to which the AI system influences patient care. An emerging gold standard—formalized across multiple academic medical centre IRB policies—is a three‐stage Framework that stages review requirements to match the risk profile of each development phase [4].

#### Stage 1: Discovery (retrospective algorithm development)

Stage 1 encompasses exploratory algorithm development on retrospective, de‐identified datasets: data curation, architecture experimentation, preliminary performance benchmarking. The critical constraint is that Stage 1 research must not impact participant healthcare, treatment or clinical decision‐making in any form.

Studies conducted on de‐identified secondary data generally qualify for Exempt or Expedited IRB review, as they represent minimal risk to participants. Oversight focuses on: verification of de‐identification adequacy, data security protocols, handling of incidental findings (e.g., incidentally detected tumours in training images) and documentation of dataset provenance. The IRB reviews only Stage 1 at this submission; additional stages require separate applications or study modifications, ensuring that IRB oversight scales appropriately as research progresses.

#### Stage 2: Silent evaluation (shadow mode translation)

The AI system processes real‐world clinical data in ‘shadow mode’—running inference in parallel with standard clinical workflows without producing outputs that enter the medical record or influence clinical decisions. Stage 2 is critical for prospective performance assessment: shadow‐mode data reflect the true deployment distribution, including case mix, acquisition variability and workflow context that retrospective training data may not capture.

Stage 2 studies typically qualify for expedited IRB review but require a formal medical device determination: the IRB must assess whether the AI system meets the regulatory definition of a medical device under code of federal regulations and Food and Drug Administration (FDA) guidance, which triggers additional oversight requirements. Systematic bias and fairness assessments across demographic and contextual subgroups are mandated as core IRB requirements for Stage 2. Safety stress‐testing with adversarial inputs (e.g., images with severe metal artefact, pathological anatomy outside the training distribution) must be documented prior to Stage 3 application.

#### Stage 3: Prospective/Interventional deployment

The AI system's outputs directly inform or execute medical decisions: real‐time fracture detection alerts to emergency physicians; AI‐guided pedicle screw trajectory to a robotic execution system; automated implant sizing recommendations to a planning surgeon. This stage requires:

- Full Board IRB Review: The convened IRB reviews Stage 3 applications, typically in consultation with subject matter experts.
- Prospective clinical validation: A registered prospective clinical trial demonstrating safety and efficacy in the target clinical context.
- Human‐override mechanisms: Technically implemented, frictionless override of any AI recommendation by a qualified clinician—a requirement that must be documented in the IRB protocol and verified in the technical implementation.
- Decommissioning plan: Formally documented criteria under which the AI system will be suspended (performance below predefined thresholds, confirmed adverse event, IRB request).
- Adverse event reporting: A documented protocol specifying actions when AI misclassification contributes to patient harm or near‐miss events.

### Regulatory frameworks: GDPR, HIPAA, the EU AI Act and SaMD

The GDPR mandates Privacy by Design (PbD) as an architectural constraint: Privacy safeguards must be integrated into the AI system from the earliest design stage, not retrofitted post hoc.

Clinical decision‐support tools that analyse patient data to support diagnostic or therapeutic decisions are classified as High‐Risk AI Systems under the European Union (EU) AI Act. This classification mandates:

- Article 9 (Risk Management System): A documented risk management system maintained continuously across the AI system's entire operational lifecycle—not as a one‐time pre‐deployment exercise.
- Article 12 (Record‐keeping): Logs capable of reconstructing system behaviour over an appropriate retention period, including timestamps, model version identifiers and inference inputs.
- Article 13 (Transparency): Documentation enabling clinicians to interpret AI outputs and understand system capabilities and limitations.
- Article 14 (Human Oversight): Technically implemented mechanisms for qualified clinicians to override, suspend or intervene in AI operation without procedural friction.
- Article 17 (Quality Management System): An integrated quality management process spanning training data documentation, performance evaluation and post‐market surveillance.

Formal AI Act conformity additionally requires a conformity assessment procedure, CE marking and registration in the EU database of high‐risk AI systems—procedural steps requiring regulatory counsel and notified body involvement that must be planned from project inception if clinical deployment is the target outcome.

For HIPAA compliance, any AI tool processing PHI must be reviewed by the institutional InfoSec Data Sharing Committee prior to data ingestion (3.3). Vendors providing AI tools for clinical use must supply written documentation on: data retention schedules; incident response procedures; audit log architecture; and compliance with the HIPAA Security Rule's technical safeguards.

Medical AI systems that meet the definition of a medical device—software intended for a medical purpose, specifically diagnosis, treatment or prevention of disease—are regulated as SaMD by the FDA (USA) and as a medical device under EU MDR 2017/745 (Europe).

FDA SaMD Classification: The FDA uses a two‐dimensional risk matrix: the severity of the condition (non‐serious, serious or critical) combined with the significance of the information the AI provides to clinical management (informing, driving or treating/diagnosing). A tool that flags a region of interest for radiologist review carries lower regulatory burden than one that autonomously determines a diagnosis.

Predetermined Change Control Plan (PCCP): The FDA allows developers to implement planned model updates (retraining, architecture modifications within documented parameters) without a new 510(k) submission per update, provided the changes remain within the PCCP's documented scope. This mechanism is essential for organizations implementing continual learning strategies, as it provides a regulatory pathway for iterative refinement without per‐version clearance overhead.

Algorithm Change Protocol: For adaptive or continuously learning systems, regulatory guidance mandates a documented Algorithm Change Protocol specifying: retraining criteria (what data distributions or performance thresholds trigger retraining); validation steps required after any model update before re‐deployment; rollback procedures; and clinician/end‐user notifications when performance shifts are detected.

Post‐Market Surveillance: The EU AI Act Article 9 requires high‐risk AI systems to maintain post‐deployment monitoring plans continuously assessing performance, safety and compliance indicators. Vendors should provide real‐world performance dashboards including sensitivity, specificity and calibration metrics disaggregated by demographic category and must implement mandatory adverse‐event reporting when misclassification contributes to patient harm or near‐miss events.

### The ‘black box’ phenomenon and explainable AI

The opacity of deep learning models presents specific challenges in orthopedic surgery: where millimetre‐scale precision is consequential, the inability to audit a model's decision pathway undermines surgeon trust and compromises the validity of patient informed consent [16]. GDPR Article 22 mandates meaningful explanations of automated decisions; the EU AI Act Article 13 mandates transparency documentation—both requirements that necessitate technically implemented explainability, not merely narrative descriptions.

Three Explainable AI (XAI) frameworks are common in orthopedic clinical contexts, each suited to different model types and data modalities:

(1) Saliency heatmaps overlaid directly on imaging data by backpropagating classification gradients to the final convolutional layer. The resulting visualization highlights anatomical regions—a specific fracture line, a periprosthetic lucency, an area of cartilage loss—that most influenced the model's decision.
(2) SHAP (SHapley Additive exPlanations): A model‐agnostic technique that quantifies the contribution of each input feature to a specific prediction using cooperative game‐theoretic Shapley values [11]. SHAP assigns each feature an additive importance score that is consistent, locally accurate and guaranteed to sum to the model output. SHAP is particularly valuable for tabular clinical risk models incorporating patient demographics, bone mineral density, prior surgical history, implant specifications and complication variables.
(3) LIME (Local Interpretable Model‐agnostic Explanations): Generates locally interpretable approximations of complex model behaviour around individual predictions by fitting a simpler, interpretable model (typically linear regression) to the local input neighbourhood [24].

Fairness monitoring: Beyond individual‐prediction explainability, systematic fairness auditing requires computing performance metrics (precision, recall, F1 score, area under the curve [AUC]) separately for protected demographic subgroups and configuring alert thresholds when performance gaps exceed tolerance.

Informed consent redesign. For Stage 3 deployment of the IRB framework for AI Research, a standard clinical consent form is often insufficient. Informed consent must be redesigned as a ‘Transparency Statement’ that explains: the specific role of the AI in the clinical pathway; the model's validated performance range and known failure modes; and the specific risks unique to AI systems—hallucination (for LLM‐based tools), algorithmic bias across demographic subgroups and model drift over time.

### Privacy‐preserving architectures: Federated learning (FL) and trusted research environments

Where multi‐institutional data sharing is precluded by GDPR, HIPAA or institutional policy, FL enables collaborative model training without transferring raw patient data [8, 20, 22, 25]. In FL, the model travels to the data rather than the data travelling to the model: each participating institution trains on local data and shares only gradient updates or model weight aggregates with a central aggregation server, keeping raw imaging behind institutional firewalls.

## PILLAR D: TECHNOLOGY AND AI ARCHITECTURES

### AI architecture selection

The choice of model architecture must be driven by the data modality, dataset scale, task complexity, inference speed requirements and interpretability constraints of the deployment context. Three architectural families are relevant to orthopedic AI.

#### Convolutional neural networks (CNNs)

CNNs remain the dominant architecture for 2D radiographic analysis, bone segmentation and implant identification; however, database size is crucial.

Given the limited size of most orthopedic datasets relative to the parameter counts of modern CNN backbones, pre‐training on large‐scale datasets (ImageNet or domain‐specific datasets such as MURA) followed by fine‐tuning on orthopedic data is the standard approach. Full fine‐tuning of all layers risks catastrophic forgetting and overfitting on small datasets. LoRA provides a parameter‐efficient alternative, enabling deployment in compute‐constrained clinical environments [6].

#### Vision transformers (ViTs)

ViTs apply multi‐head self‐attention mechanisms across image patches, capturing global spatial dependencies across the entire field of view in a single forward pass—overcoming the local receptive field constraint inherent in convolutional operations. ViTs are emerging as the preferred architecture for complex orthopedic classification tasks requiring reasoning about relationships between anatomically distinct structures [15].

The attention maps of ViTs provide a form of built‐in interpretability: attention weights across patches can be visualized as spatial importance maps analogous to Gradient‐weighted Class Activation Mapping (Grad‐CAM) outputs, supporting the XAI transparency requirements of the EU AI Act.

Hybrid architectures combining CNN feature extractors with Transformer encoders (e.g., using a ResNet backbone to generate patch embeddings fed into a Transformer encoder) often outperform pure ViTs on smaller datasets by leveraging the inductive bias of CNNs while retaining global reasoning capabilities for complex multi‐site tasks.

#### LLMs, NLP and agentic AI

Clinical NLP applications. LLMs address the unstructured text dimension of orthopedic data: operative notes, radiology reports, discharge summaries and patient‐reported outcome narratives.

RAG grounds LLM outputs in an indexed knowledge base of authoritative orthopedic sources, reducing hallucinations while providing traceable citations [9]. RAG achieves near‐zero hallucination rates in some implementations, but introduces latency overhead, rendering it unsuitable for real‐time intraoperative decision support where sub‐second responses are critical.

Agentic AI architectures. The maturity model of clinical AI is shifting from passive information retrieval to agentic systems capable of chain‐of‐thought reasoning and tool use [17]. An agent, in this context, is an entity that perceives its environment (patient records, imaging findings, laboratory results), reasons about its context and takes actions to achieve a clinical goal.

The MAI‐Dx orchestration framework coordinates multiple specialized LLM agents—hypothesis formulation, checklist validation, differential diagnosis ranking—in a ‘chain‐of‐thought debate’ that mimics multi‐specialty case conference [13]. The critical governance constraint is that as systems become more autonomous, the architectural requirement shifts from pure accuracy to explainability and auditability, with clinicians retaining the ‘human‐in‐the‐loop’ role before any autonomous action is executed.

## CONCLUSION

A successful AI‐driven orthopedic research project requires the simultaneous and coordinated satisfaction of four prerequisite domains. The specific technical requirements that remain most frequently unmet in current practice are: (1) product‐centric team topologies that preserve domain expert cognitive resources for clinical validation tasks; (2) interoperability with bulk data exports for scalable dataset assembly; (3) de‐identification pipelines that preserve clinical semantics while removing PHI; (4) guideline compliant reporting with external validation; (5) IRB applications structured according to the three‐stage framework that scales oversight to deployment risk.

The persistent gap between publication volume and clinical deployment rate is not primarily an algorithmic failure. It reflects an engineering and organizational failure to satisfy the prerequisites described in this article from project inception. The frameworks provided here constitute a practical specification for research groups committed to producing orthopedic AI systems that are not merely accurate in retrospective studies, but operational, safe and governable in the clinical environments where they are ultimately needed.

## AUTHOR CONTRIBUTIONS

Umile Giuseppe Longo, Mario Merone, Emiliano Schena, Benedetta Bandini, Guido Nicodemi, Bálint Zsidai, Ann‐Sophie Hilkert and Felix C. Oettl performed the main manuscript preparation and literature review. All remaining authors (Eric Hamrin Senorski, Alberto Grassi, Christophe Ley, Elmar Herbst, Michael T. Hirschmann, Sebastian Kopf, Romain Seil, Thomas Tischer, Robert Feldt and Kristian Samuelsson) critically reviewed the manuscript and contributed to its final preparation. All authors read and approved the final manuscript.

## CONFLICT OF INTEREST STATEMENT

Kristian Samuelsson is a member of the Board of Directors of Getinge AB (publ) and a medtech advisor to Carl Bennet AB. Felix C. Oettl has received speaker fees from OPED AG. The remaining authors declare no conflict of interest.

## ETHICS STATEMENT

The authors have nothing to report.
